# Heme protects *Pseudomonas aeruginosa* and *Staphylococcus aureus* from calprotectin-induced iron starvation

**DOI:** 10.1074/jbc.RA120.015975

**Published:** 2020-12-09

**Authors:** Emily M. Zygiel, Adunoluwa O. Obisesan, Cassandra E. Nelson, Amanda G. Oglesby, Elizabeth M. Nolan

**Affiliations:** 1 Department of Chemistry, Massachusetts Institute of Technology, Cambridge, Massachusetts, USA; 2 School of Pharmacy, Department of Pharmaceutical Sciences, University of Maryland, Baltimore, Maryland, USA; 3 School of Medicine, Department of Microbiology and Immunology, University of Maryland, Baltimore, Maryland, USA

**Keywords:** calprotectin, heme, *Pseudomonas aeruginosa*, *Staphylococcus aureus*, iron, innate immunity, nutritional immunity, CAS, chrome azurol S, CDM, chemically defined medium, CF, cystic fibrosis, CP, calprotectin, Fe(II), ferrous, Fe(III), ferric, Fur, ferric uptake regulator inserted, Has, heme assimilation system, ICP-MS, inductively coupled plasma–mass spectrometry, Isd, iron-regulated surface determinants, KO, knockout, PCA, phenazine-1-carboxylate, Phu, Pseudomonas heme uptake, PYO, pyocyanin, TSB, tryptic soy broth

## Abstract

*Pseudomonas aeruginosa* and *Staphylococcus aureus* are opportunistic bacterial pathogens that cause severe infections in immunocompromised individuals and patients with cystic fibrosis. Both *P. aeruginosa* and *S. aureus* require iron to infect the mammalian host. To obtain iron, these pathogens may rely on siderophore-mediated ferric iron uptake, ferrous iron uptake, or heme uptake at different points during infection. The preferred iron source depends on environmental conditions, including the presence of iron-sequestering host-defense proteins. Here, we investigate how the presence of heme, a highly relevant iron source during infection, affects bacterial responses to iron withholding by the innate immune protein calprotectin (CP). Prior work has shown that *P. aeruginosa* is starved of iron in the presence of CP. We report that *P. aeruginosa* upregulates expression of heme uptake machinery in response to CP. Furthermore, we show that heme protects *P. aeruginosa* from CP-mediated inhibition of iron uptake and iron-starvation responses. We extend our study to a second bacterial pathogen, *S. aureus*, and demonstrate that CP also inhibits iron uptake and induces iron-starvation responses by this pathogen. Similarly to *P. aeruginosa*, we show that heme protects *S. aureus* from CP-mediated inhibition of iron uptake and iron-starvation responses. These findings expand our understanding of microbial responses to iron sequestration by CP and highlight the importance of heme utilization for bacterial adaptation to host iron-withholding strategies.

*Pseudomonas aeruginosa* and *Staphylococcus aureus* are two of the most prevalent nosocomial pathogens that can cause deleterious infections in part due to the emergence of multidrug-resistant strains (1). These pathogens frequently colonize the same sites, including the urinary tract, burn and surgical wounds, and the upper respiratory tract. In patients with the hereditary lung disease cystic fibrosis (CF), *P. aeruginosa* and *S. aureus* are the most prevalent bacterial pathogens that colonize the lung, and their co-colonization is associated with poor patient outcomes (2). The virulence of these two pathogens in the CF lung and severity of CF lung disease are closely linked to iron homeostasis (3). In particular, the anti-staphylococcal activity of *P. aeruginosa* has been shown to be highly dependent on iron availability (4). Moreover, as an essential nutrient, iron also plays critical roles in the survival of these organisms and regulates the production of several virulence factors that alter their interactions with the host (5, 6, 7). To support their iron requirements during infection, both *P. aeruginosa* and *S. aureus* use several mechanisms to scavenge different forms of iron in the host environment.

*P. aeruginosa* has several iron-uptake strategies to acquire iron from the host, including siderophore-mediated ferric [Fe(III)] uptake, ferrous [Fe(II)] uptake, and heme uptake. *P. aeruginosa* produces two siderophores, pyoverdine and pyochelin, to obtain Fe(III) in the host (8). Fe(II) can be prevalent in low-oxygen or reducing environments, and *P. aeruginosa* uses the Feo Fe(II) iron-uptake system to acquire iron in these environments (9). In addition, a class of redox-cycling molecules called phenazines can facilitate Fe(II) uptake by reducing extracellular Fe(III) to Fe(II), which allows for more efficient Fe(II) uptake by the Feo system (10). To acquire heme, *P. aeruginosa* uses a hemophore-dependent heme assimilation system (Has) and a non-hemophore-dependent heme uptake system (Pseudomonas heme uptake [Phu]) (11). The Has system uses the secreted hemophore HasAp to capture extracellular heme, which is then taken up by an outer membrane TonB-dependent receptor, whereas the Phu system uses a TonB-dependent receptor to directly sequester heme (12). Prior work examining CF lung sputum samples suggested that *P. aeruginosa* switches from using siderophores to depending more heavily on heme-acquisition and Fe(II)-acquisition strategies as infection progresses (13, 14).

Two iron-acquisition strategies that are important for *S. aureus* and have been extensively characterized are siderophore-mediated Fe(III) uptake and heme uptake (5). *S. aureus* produces the siderophores staphyloferrin A and B, which allow the bacterium to compete for Fe(III) with host proteins lactoferrin and transferrin (15). Although these siderophores provide an important iron-acquisition strategy for *S. aureus*, studies have shown that *S. aureus* prefers heme, which accounts for ∼80% of the iron in the host, as an iron source (5, 16). *S. aureus* is able to access heme by secreting hemolysins, which lyse erythrocytes to release hemoglobin (5). Heme-uptake systems, including the iron-regulated surface determinants (Isd) transport system, subsequently import free heme into the bacterium (5). Prior work has shown that *S. aureus* preferentially uses heme and switches to using siderophores only when heme is less available (16). The importance of Fe(II) uptake for *S. aureus* is not understood, but a putative metal uptake system in *S. aureus* that has homology to other bacterial Feo transporters has been identified and shown to be upregulated in iron-deficient growth conditions (17).

In response to bacterial infection, the host uses iron-sequestering host-defense proteins, which are capable of limiting Fe(III), Fe(II), and heme at infection sites. These proteins include lactoferrin, lipocalin-2, haptoglobin, hemopexin, and calprotectin (CP) and serve to starve invading bacterial pathogens of iron as part of the metal-withholding innate immune response (12, 18, 19, 20, 21). CP (S100A8/S100A9 hetero-oligomer, MRP9/MRP14 oligomer) is a host-defense protein that sequesters divalent first-row transition metal ions, including Mn(II), Fe(II), Ni(II), and Zn(II) (20, 21, 22, 23). CP is released from white blood cells at infection sites, where it is capable of competing with microbial pathogens for nutrient metal ions (23). Prior work revealed a broad-spectrum antimicrobial activity of CP, which is attributed to its ability to chelate multiple nutrient metal ions and thereby prevent microbial acquisition of these nutrients (20, 23, 24, 25). From the standpoint of iron in infection and immunity, CP is the only known Fe(II)-sequestering host-defense protein (20, 21, 22).

In the current work, we evaluate how the presence of heme affects responses of *P. aeruginosa* and *S. aureus* to Fe(II) withholding by CP. This topic is of significant interest because CP colocalizes with *P. aeruginosa* and *S. aureus* in the CF lung and its levels correlate with disease severity (26, 27). Importantly, this study represents a step toward addressing the chemical complexity of infection sites by evaluating how a biologically relevant iron source affects bacterial responses to Fe(II) sequestration by CP. We report that *P. aeruginosa* upregulates expression of heme-uptake machinery in response to CP. Furthermore, we demonstrate that heme alleviates the inhibition of *P. aeruginosa* iron uptake and iron-starvation responses that CP causes (Fig. 1). We also examine the effect of CP on *S. aureus* iron homeostasis, providing the first comprehensive evaluation of whether CP induces iron starvation in this bacterial pathogen. We report that eight *S. aureus* strains, including CF clinical isolates, exhibit decreased iron uptake after CP treatment. Moreover, our results uncover that CP induces iron-starvation responses in *S. aureus*. Similarly to our observations for *P. aeruginosa*, heme protects *S. aureus* from both inhibition of iron uptake and iron starvation by CP. Taken together, these findings reveal the importance of heme for adaptation of *P. aeruginosa* and *S. aureus* to Fe(II) withholding by CP.

**Figure 1:**
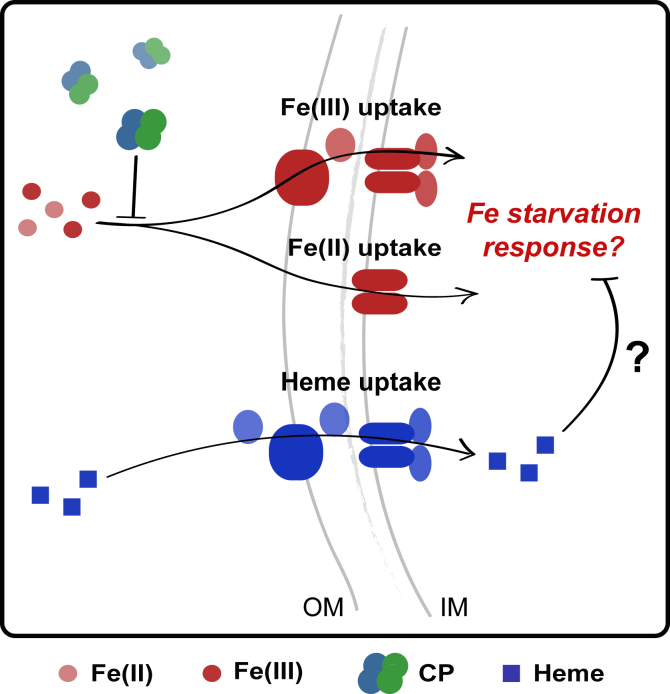
**Overview of working model depicted for *Pseudomonas aeruginosa*.** This model is also investigated for *Staphylococcus aureus*. CP inhibits iron uptake and induces iron-starvation responses in the absence of heme. Here, we test the hypothesis that heme, an alternative iron source, will protect *P. aeruginosa* and *S. aureus* from Fe starvation induced by CP. CP, calprotectin; IM, inner membrane; OM, outer membrane.

## Results

### *P. aeruginosa* upregulates expression of heme utilization machinery in response to CP

**Figure 2:**
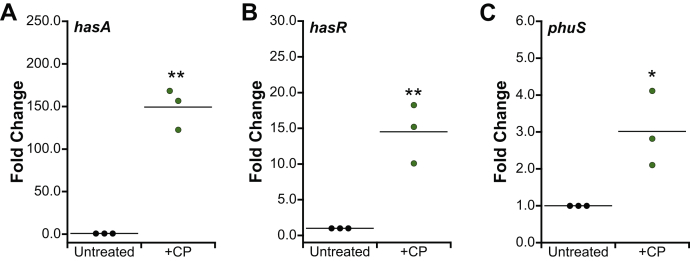
**RT-PCR analysis of *Pseudomonas aeruginosa hasA*, *hasR*, and *phuS.*** PA14 and PAO1 were grown in the absence or presence of CP (10 μM) in CDM. mRNA levels were normalized to *oprf*, and the fold change relative to the untreated condition for PA14 *hasA* (*A*) and *hasR* (*B*), and PAO1 *phuS* (*C*) is presented (N = 3, ∗*p* < 0.05, ∗∗*p* < 0.01). CDM, chemically defined medium; CP, calprotectin.

*P. aeruginosa* uses heme uptake machinery during infection, and in some cases, preferentially utilizes heme over other iron sources (14, 28). Because CP can sequester Fe(II) and thereby lower the availability of non-heme iron, we questioned whether *P. aeruginosa* resists CP by upregulating expression of heme-acquisition machinery to obtain heme iron. We analyzed the effect of CP on transcriptional changes in *hasA* and *hasR*, two components of the Has heme uptake system, and *phuS*, a component of the Phu heme uptake system. Expression of *hasA*, *hasR*, and *phuS* was increased in cultures grown in the presence of CP (Fig. 2), indicating that *P. aeruginosa* upregulates heme-acquisition machinery in response to CP. Based on this observation, we reasoned that *P. aeruginosa* may adapt to CP by utilizing heme to rescue cellular iron levels.

### Heme allows *P. aeruginosa* to prevent depletion of cellular iron by CP

**Figure 3:**
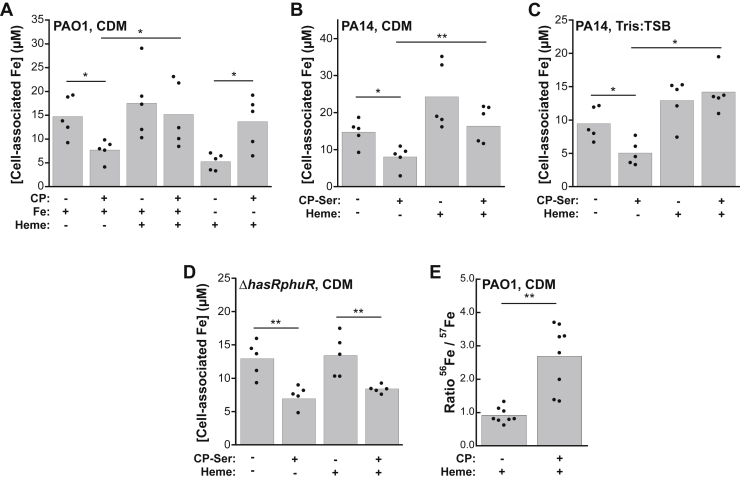
**Heme allows *Pseudomonas aeruginosa* to prevent depletion of cellular iron by CP.***A*, cell-associated iron in *P. aeruginosa* PAO1 grown in CDM or iron-depleted CDM in the absence or presence of CP (10 μM) and heme (5 μM) (N = 5, ∗*p* < 0.05). *B* and *C*, cell-associated iron for *P. aeruginosa* PA14 grown in CDM (*B*) or Tris:TSB (*C*) in the absence or presence of CP-Ser (10 μM) and heme (5 μM) (N = 5, ∗*p* < 0.05, ∗∗*p* < 0.01). *D*, cell-associated Fe for *P. aeruginosa* PAO1 Δ*hasRphuR* grown in CDM in the absence or presence of CP-Ser (10 μM) and heme (5 μM) (N = 5, ∗∗*p* < 0.01). *E*, cell-associated ratio of ^56^Fe:^57^Fe in *P. aeruginosa* PAO1 grown in CDM in the absence or presence of CP (10 μM) and heme (5 μM) (N = 8, ∗∗*p* < 0.01). CDM, chemically defined medium; CP, calprotectin.

We analyzed cellular metal inventory by inductively coupled plasma–mass spectrometry (ICP–MS) to determine the effect of heme supplementation on iron uptake during growth of *P. aeruginosa* in the absence or presence of CP. These studies were performed with CP and/or CP-Ser, which is a CP variant with two Cys→Ser point mutations [S100A8(C42S)/S100A9(C3S)]. CP-Ser was originally used in coordination chemistry studies to avoid potential complications of the two Cys residues (25). It has been commonly used in metal-binding and antimicrobial studies of CP and displays comparable activity to CP (25). *P. aeruginosa* PAO1 and PA14 cultures were grown in a chemically-defined medium (CDM) with or without a 5 μM heme supplement. We chose to perform these studies using CDM, which contains ∼300 nM manganese, ∼5 μM iron, and ∼6 μM zinc because it is a defined medium that lacks heme (29). We also performed select experiments with Tris:tryptic soy broth (TSB), which contains ∼150 nM manganese, ∼4 μM iron, and ∼5 μM zinc (Table S1). This undefined medium contains low levels of heme but serves as a point of comparison based on its use in prior studies of CP and *P. aeruginosa* (22). Regardless of the medium composition, we observed a decrease in cell-associated iron content for cultures of both PAO1 and PA14 that were grown in the presence of CP, in agreement with our prior work (Fig. 3, *A*–*C*) (22). In contrast, for cultures grown in the presence of both heme and CP, we observed iron levels comparable with the untreated cultures and the cultures treated with only heme (Fig. 3, *A*–*C*). These data suggest that when heme is available, *P. aeruginosa* will use it as an iron source and thereby resist the consequences of Fe(II) withholding by CP (Fig. 3, *A*–*C*). In addition, when we used a heme-uptake knockout (KO) strain of *P. aeruginosa* PAO1 (Δ*hasRphuR*) in this experiment, we found that the presence of heme did not affect inhibition of iron uptake by CP when heme could not be used (Fig. 3*D*). Cell-associated manganese for PA14 grown in Tris:TSB was also reduced by CP, consistent with our prior results for PAO1 in this medium (Fig. S3*A*) (22). For PAO1 and PA14 grown in CDM, CP did not reduce manganese uptake, indicating that medium conditions may affect manganese withholding by CP (Figs. S1*A* and S2*A*). We observed negligible changes in cell-associated nickel, copper, and zinc in PAO1 and PA14 from CP treatment, consistent with our prior study (Figs S1, *C–E*, S2, *C–E*, and S3, *C–E*) (22). The presence of heme did not alter the effect of CP on cell-associated manganese, nickel, copper, or zinc in either CDM or Tris–TSB (Figs. S1, *A* and *C–E*, S2, *A* and *C–E*, and S3, *A* and *C–E*).

We next questioned how CP affects cellular iron uptake if heme is the only iron source available to *P. aeruginosa*. We grew *P. aeruginosa* PAO1 in CDM prepared without iron salt and supplemented with 5 μM heme in the absence or presence of CP and measured cell-associated iron levels. When heme was the only iron source, CP treatment afforded an increase in iron uptake relative to the heme-only condition (Fig. 3*A*). We reason that the increase in iron uptake by *P. aeruginosa* when grown with CP and heme is attributable to the enhanced expression of heme uptake machinery caused by CP (Fig. 2).

To probe whether heme uptake accounted for the observed increase in cell-associated iron in cultures treated with heme and CP, we used the stable ^57^Fe isotope to prepare CDM, allowing us to simultaneously track the uptake of heme iron (^56^Fe; 91.75% natural abundance) and non-heme iron (^57^Fe; 95% isotopic purity) using ICP–MS. For cultures treated with heme and CP, we observed a significantly higher ^56^Fe:^57^Fe ratio (∼2.7) than cultures treated with heme alone (∼1.0) (Fig. 3*E*). Thus, when CP is present in culture, *P. aeruginosa* prioritizes heme as a source of iron.

### CP-induced iron-starvation responses in *P. aeruginosa* are attenuated when heme is available

Building upon our current observations, we hypothesized that heme utilization protects *P. aeruginosa* from undergoing CP-induced iron-starvation responses. We therefore examined select markers of iron-starvation responses in *P. aeruginosa* cultures treated with CP and/or heme.

**Figure 4:**
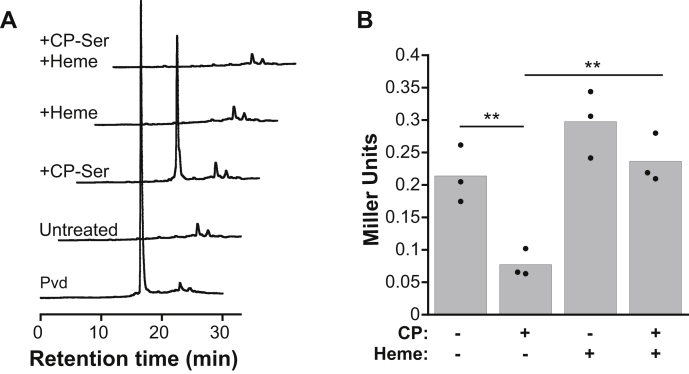
**Heme protects *Pseudomonas aeruginosa* from CP-mediated iron-starvation responses.***A*, fluorescence intensity detected by HPLC (λ_ex_ = 398 nm, λ_em_ = 455 nm) for pyoverdine (Pvd) standard and supernatants from PA14 grown in CDM in the absence or presence of CP-Ser (10 μM) and heme (5 μM). Pvd standard was purified as described previously (22). *B*, PAO1 *antR* translation was measured using an *antR-lacZ* translational fusion (*Pseudomonas aeruginosa* PAO1/P_*antR*_-‘*lacZ*^*-*SD^) following growth in Tris:TSB in the absence or presence of CP (10 μM) and heme (5 μM). β-Galactosidase activity was assayed in cell suspensions (N = 3, ∗∗*p* < 0.01). CDM, chemically defined medium; CP, calprotectin.

*P. aeruginosa* upregulates production of the siderophore pyoverdine in response to CP (22). We analyzed pyoverdine levels in culture supernatants grown in the absence or presence of CP-Ser and heme and found that in comparison with the cultures treated with CP-Ser where pyoverdine was observed in culture supernatants, cultures treated with CP-Ser and heme had negligible pyoverdine levels (Fig. 4*A*). These data indicate that heme prevents CP from inducing a siderophore-mediated iron-starvation response.

*P. aeruginosa* also downregulates expression of *antR*, which encodes an activator of an anthranilate catabolic pathway that is regulated by iron via ferric uptake regulator (Fur)-controlled PrrF sRNAs, in response to CP (22). AntR directly controls transcription of iron-containing proteins; thus, when iron availability is low, PrrF sRNAs inhibit *antR* translation as part of an iron-sparing response (30). We evaluated activity of an *antR* translational reporter in *P. aeruginosa* PAO1 (PAO1/P_*antR*_-‘*lacZ*^-SD^) grown in the absence or presence of CP and heme (31). We observed lower levels of *antR* reporter activity in cultures grown in the presence of CP than in its absence, in agreement with our prior work (22). In cultures grown in the presence of heme and CP, the observed levels of *antR* reporter activity were comparable with those of the untreated and heme-treated cultures and significantly higher than those of CP-treated cultures (Fig. 4*B*). Thus, the presence of heme prevents CP from inducing a PrrF-regulated iron-starvation response.

### Heme does not alter the effect of CP on *P. aeruginosa* phenazine production

**Figure 5:**
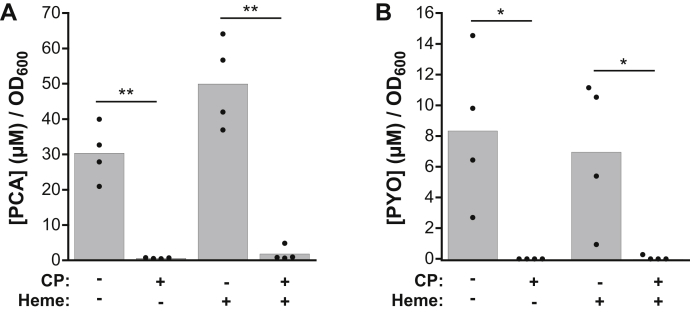
**Heme does not affect inhibition of phenazine production by CP.** Average (*A*) PYO and (*B*) PCA concentrations in supernatants from *Pseudomonas aeruginosa* PA14 cultures grown in CDM in the absence or presence of CP-Ser (10 μM) and heme (5 μM) (N = 4, ∗*p* < 0.05, ∗∗*p* < 0.01). CDM, chemically defined medium; CP, calprotectin; PCA, phenazine-1-carboxylate; PYO, pyocyanin.

The redox-cycling function of phenazines serves a number of purposes for *P. aeruginosa* biology, including promotion of Fe(II) uptake by the Feo system (10). We previously found that both CP treatment and iron-deficient conditions inhibit phenazine production by *P. aeruginosa* (22). Based on our current results, we questioned whether heme utilization would recover phenazine production by *P. aeruginosa* in the presence CP. We quantified the concentrations of two phenazines, phenazine-1-carboxylate (PCA) and pyocyanin (PYO), in supernatants of *P. aeruginosa* PA14 cultures grown in the absence or presence of heme and CP-Ser (Fig. 5). In agreement with our prior data (22), we observed a significant decrease in levels of both PCA and PYO for cultures treated with CP-Ser (Fig. 5, *A* *and* *B*). For cultures grown in the presence of heme, CP-Ser caused a similar decrease in phenazine levels. This result differs from the heme-mediated recovery of siderophore- and PrrF-controlled iron-starvation responses caused by CP. It is possible that CP affects distinct regulatory systems that inhibit phenazine production even when other iron-regulated responses are recovered by heme. Indeed, phenazine production can be modulated by a number of signals, including zinc- and quorum-sensing molecules (32, 33). Iron regulation in *P. aeruginosa* is complex (6), and these results indicate that its response to CP is also complex. Furthermore, different iron sources may be sourced or sensed differently in the cell, and it is possible that while heme can supply iron that alleviates some iron-starvation responses, it is not able to recover phenazine production.

### CP inhibits iron uptake and induces iron starvation in *S. aureus*

Our findings in *P. aeruginosa* prompted us to evaluate how heme affects the response of another bacterial pathogen to CP. We chose *S. aureus* because of its clinical relevance to coinfection with *P. aeruginosa*, particularly in the CF lung, as well as the importance of heme for its pathogenesis (5). The role of heme in *S. aureus* pathogenesis has been extensively studied, and heme has been shown to be the preferred iron source during *S. aureus* infection (16). Moreover, *S. aureus* often co-colonizes the CF lung with *P. aeruginosa* when CP is present and therefore may compete for available iron sources. Prior work on CP and *S. aureus* has largely focused on Mn(II) withholding (24, 34, 35, 36, 37, 38). Consequently, the prevailing dogma is that the antibacterial activity of CP against *S. aureus* is primarily a result of Mn(II) starvation. Indeed, it has been assumed that CP does not affect iron homeostasis in *S. aureus* (24, 34). Based on the growing appreciation of CP to limit Fe(II), we aimed to evaluate the impact of CP on *S. aureus* iron homeostasis pathways.

**Figure 6:**
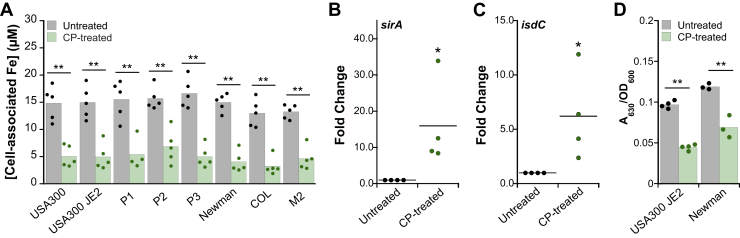
**CP treatment reduces iron uptake and promotes siderophore production by *Staphylococcus aureus***. *A*, cell-associated iron for *S. aureus* strains (Table S2) grown in LB medium in the absence or presence of CP (20 μM) (N = 5, ∗∗*p* < 0.01). *B* and *C*, RT-PCR analysis of *S. aureus sirA* (*B*) and *isdC* (*C*) mRNA levels in USA300 JE2 grown in the absence or presence of CP (20 μM). mRNA levels were normalized to *sigA*, and the fold change relative to the untreated condition is presented (N = 4, ∗*p* < 0.05). *D*, the CAS assay results for *S. aureus* USA300 JE2 and Newman supernatants from cultures grown in LB medium in the absence or presence of CP (20 μM). A_630_ measurements were normalized to culture absorbance at 600 nm (N ≥ 3, ∗∗ *p* < 0.01). CAS, chrome azurol S; CDM, chemically defined medium; CP, calprotectin.

We previously reported that CP inhibits iron uptake by *S. aureus* USA300 JE2 cultured in LB medium (22) and sought to determine whether this observation is generalizable to other *S. aureus* strains. We screened eight *S. aureus* strains (Table S2), including three clinical isolates from patients with CF, for the effect of CP on metal uptake during growth in LB medium. For all strains tested, we observed a ∼60% reduction in cell-associated iron levels when cells were grown in the presence of CP (Fig. 6*A*). In addition, for all strains tested, we observed reduction of cell-associated manganese and copper levels, but not nickel or zinc under these conditions (Fig. S4). Thus, strain background appears to have a negligible effect on inhibition of *S. aureus* metal uptake by CP, at least under these experimental conditions.

We next questioned whether CP induces iron-starvation responses in *S. aureus*, as has been previously observed for *P. aeruginosa* and *Acinetobacter baumannii* (22, 39). When confronted with iron limitation, *S. aureus* Fur is in the apo form and thus expression of genes involved in iron homeostasis, such as siderophore biosynthesis and transport machinery and heme uptake machinery, are derepressed (5). We analyzed the effect of CP on expression of *sirA*, which encodes for the solute-binding protein SirA that binds staphyloferrin B, and *isdC*, which encodes for the heme-transport protein IsdC. Expression of both *sirA* and *isdC* was upregulated in the presence of CP, indicating a CP-induced iron-starvation response (Fig. 6, *B* and *C*).

To determine whether treatment of cultures with CP increased siderophore production, we measured siderophore levels in culture supernatants from *S. aureus* USA300 JE2 and *S. aureus* Newman grown in the absence or presence of CP using a chrome azurol S (CAS) assay (40). The CAS assay monitors the removal of Fe(III) bound to the CAS dye by siderophores, which results in a change in the optical absorption features of the dye that can be measured at 630 nm. We first determined that the addition of CP to CAS in the buffered solution or under the assay conditions had negligible effect on the A_630_ value, indicating that CP neither removes Fe(III) from the CAS dye (Fig. S5*A*) nor affords a positive CAS result (Fig. S5*B*). For cultures grown in the presence of CP, a significantly lower A_630_ was observed in comparison with untreated cultures (Fig. 6*D*), revealing removal of bound Fe(III) from the CAS dye. Thus, CP induces siderophore production by *S. aureus*, indicating a siderophore-mediated iron-starvation response as previously observed for *P. aeruginosa* and *A. baumannii* (22, 39).

### Heme protects *S. aureus* from inhibition of iron uptake and iron starvation

**Figure 7:**
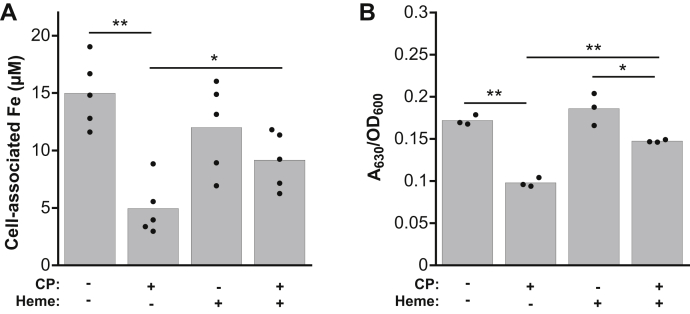
**Heme protects*Staphylococcus aureus*from depletion of cellular iron and induction of iron starvation by CP**. *A*, cell-associated iron for *S. aureus* USA300 JE2 grown in LB medium in the absence or presence of CP (20 μM) and heme (5 μM) (N = 5, ∗*p* < 0.05, ∗∗ *p* < 0.01). *B*, the CAS assay for *S. aureus* USA300 JE2 supernatants from cultures grown in LB medium in the absence or presence of CP (20 μM) and heme (5 μM). A_630_ measurements were normalized to culture absorbance at 600 nm (N = 3, ∗*p* < 0.05, ∗∗*p* < 0.01). CAS, chrome azurol S; CP, calprotectin.

The enhanced expression of *isdC* in CP-treated cultures of *S. aureus* led us to question whether heme protects *S. aureus* from iron starvation by CP. When choosing a medium to evaluate the effect of heme on the response of *S. aureus* to CP, we first attempted to use CDM, as we did for the experiments with *P. aeruginosa* described above, because it lacks heme. However, when *S. aureus* was grown in CDM containing ≥2.5 μM heme, negligible growth occurred. Indeed, heme can be toxic to Gram-positive bacteria such as *S. aureus*. In prior literature, ≤5 μM heme was shown to be tolerated, albeit in a richer medium (41). Thus, we performed these analyses in LB medium because (i) heme treatment caused less toxicity in this medium (Table S4) and (ii) our prior studies indicated that CP inhibits *S. aureus* iron uptake in LB medium (22). ICP–MS analysis of metal levels in LB medium indicated that the medium contains ∼300 nM manganese, ∼8 μM iron, and ∼16 μM zinc (Table S1). Analysis of cell-associated metal levels in *S. aureus* USA300 JE2 cultures grown in LB in the presence of heme and CP indicated significantly more iron uptake than those treated with CP alone (Fig. 7*A*). The presence of heme had negligible effects on the uptake of manganese, nickel, copper, and zinc by CP-treated *S. aureus* (Fig. S6), and the effect of CP on the uptake of these metals was consistent with our prior observations (22). To determine whether heme supplementation alters the CP-mediated increase in siderophore production by *S. aureus*, we analyzed siderophore levels in *S. aureus* USA300 JE2 supernatants from cultures grown in the absence or presence of CP and heme using the CAS assay. In comparison with cultures that were treated with CP, we observed a significant reduction of siderophore levels in the CP- and heme-treated cultures (Fig. 7*B*). Thus, heme protects *S. aureus* from CP-induced iron-starvation responses.

### Heme alters the growth-inhibitory activity of CP against *P. aeruginosa* and *S. aureus* cultures

**Figure 8:**
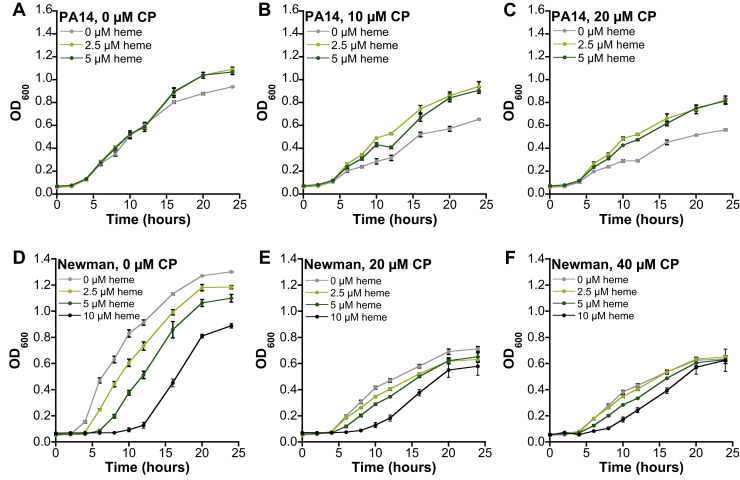
**Effect of heme and CP on the growth of *Pseudomonas aeruginosa* and *Staphylococcus aureus*.***A–C*, *P. aeruginosa* PAO1 was grown in Tris:TSB and treated with 0, 10, or 20 μM CP and 0, 2.5, or 5 μM heme. *D–F*, *S. aureus* USA300 JE2 was grown in LB medium with 0, 20, or 40 μM CP and 0, 2.5, 5, or 10 μM heme (N = 3, SEM). CP, calprotectin.

We next considered that heme may attenuate the growth-inhibitory activity of CP. We performed growth curve assays for *P. aeruginosa* and *S. aureus* cultured in the absence or presence of CP and heme. For *P. aeruginosa* PAO1 and PA14, we observed that addition of 2.5 μM or 5 μM heme to the growth medium significantly reduced the growth-inhibitory activity of CP in cultures treated with 10 μM or 20 μM CP (Figs. 8, *A*–*C* and S7, *A–C*). Heme did not promote the growth of *P. aeruginosa* PAO1 in the absence of CP (Fig. 8*A*). In contrast, growth promotion was observed for *P. aeruginosa* PA14 cultures at later time points (≥16 h) (Fig. S7*A*).

For untreated cultures of *S. aureus* USA300 JE2, we observed that all heme concentrations tested (2.5, 5, and 10 μM) resulted in growth inhibition, in agreement with prior observations of heme toxicity in *S. aureus* cultures (Figs. 8, *D* and *F* and S7, *D* and *F*) (41). However, when 20 μM or 40 μM CP was present, we observed that ≤5 μM heme had negligible effect on *S. aureus* growth (Figs. 8, *E* and *F* and S7, *E* and *F*). It is possible that heme is simultaneously reducing growth because of its toxicity and promoting growth by providing an accessible iron source during CP treatment, resulting in an overall negligible change in growth phenotype for *S. aureus* when both CP and heme are present.

## Discussion

**Figure 9:**
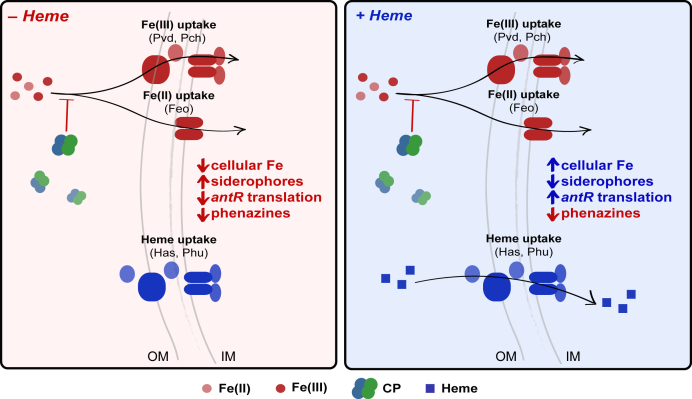
**Model for the effect of heme on CP-mediated iron starvation in *Pseudomonas aeruginosa* based on Zygiel *et al.*** (22) **and the current work.** (Left) When heme is unavailable, CP inhibits *P. aeruginosa* iron uptake and induces iron-starvation responses including increased siderophore production, inhibition of *antR* translation, and inhibition of phenazine production. (Right) When heme is available, *P. aeruginosa* can utilize heme to mitigate the effect of CP on iron uptake, which alleviates its iron-starvation responses to CP. Inhibition of phenazine production by CP is maintained in the presence of heme. Figure S8 provides a model for the effect of heme on CP-mediated iron starvation in *Staphylococcus aureus* based on the iron-starvation responses observed in this work. CP, calprotectin; IM, inner membrane; OM, outer membrane.

The current work uncovers that the availability of heme as an iron source reduces the ability of CP to starve *P. aeruginosa* and *S. aureus* of iron (Figs. 9 and S8). For *P. aeruginosa*, the importance of heme uptake has only recently been appreciated, and the current work indicates that when *P. aeruginosa* is challenged with CP, heme is an important iron source for the bacterium. For *S. aureus*, these findings integrate early work on the importance of heme uptake for *S. aureus* with recent observations of the iron-withholding capabilities of CP, providing new insight into the ability of *S. aureus* to resist CP via heme utilization.

Our results show that CP inhibits iron uptake and induces iron-starvation responses in *S. aureus* (Fig. S8). These new results advance our growing understanding for the role of CP in iron withholding from bacterial pathogens, which has been described for *P. aeruginosa* and *A. baumannii* (22, 39). In general, the effect of CP on *S. aureus* iron homeostasis has been overlooked. However, a prior study that focused on the impact of CP on *S. aureus* Zn homeostasis reported RNAseq data that show upregulation of several genes involved in siderophore-mediated iron acquisition, including *sirA*, *sirB*, *sbnC*, and *fhuB*, when *S. aureus* is cultured in the presence of CP (42). These prior data are in agreement with our current results, and together provide compelling evidence for iron withholding by CP in *S. aureus* culture, and should prompt further consideration of the effect of CP on *S. aureus* iron homeostasis *in vivo*. *S. aureus* is iron-starved in the mammalian host, as shown by studies that report upregulation of Fur-repressed genes during systemic infection of mice (43, 44). However, the specific role of CP in withholding iron from *S. aureus* during infection has not been evaluated, as prior studies of *S. aureus* infections in CP-KO mice have focused on the effects of manganese and zinc withholding (24, 35, 45).

Heme is the most abundant iron source in the host and is largely contained in erythrocytes bound to hemoglobin. Thus, many bacterial pathogens have evolved mechanisms to extract heme from host stores for subsequent import and utilization. For *S. aureus*, the Isd heme-acquisition system is an important determinant for pathogenesis. Murine models of *S. aureus* skin infection have shown decreased bacterial burdens for the Δ*isdA* strain in comparison with the parent strain Newman (46). In a systemic infection model, *S. aureus isdB* was differentially expressed in various tissues, indicating different degrees of iron starvation in distinct parts of the body, and a lower bacterial burden was observed in the heart for Δ*isdB* relative to the parent strain Newman (43). In comparison with studies in *S. aureus*, the importance of heme uptake in *P. aeruginosa* has been appreciated relatively recently. Nevertheless, several investigations have provided insight into the importance of *P. aeruginosa* heme-uptake systems during infection. A study that analyzed characteristics of *P. aeruginosa* growth in human burn wound exudate showed that genes for heme uptake and metabolism (*phu* genes and *hemO*) were upregulated in comparison with LB medium (47). A separate study comparing gene expression between *in vitro* and clinical samples of *P. aeruginosa* indicated that genes for heme uptake (*phuR and phuS*) were more highly expressed in the clinical samples, suggesting that heme uptake is important for *P. aeruginosa* survival in the human host (48). During lung infection, in particular, recent work demonstrated that as *P. aeruginosa* adapts to the host environment, it becomes highly dependent on heme utilization (14, 28). Furthermore, in a murine model of lung infection, *P. aeruginosa* PAO1 Δ*hasR* achieved a lower bacterial burden than the parent strain PAO1, indicating the importance of the Has heme uptake system for successful colonization of *P. aeruginosa* in the murine lung (49). Despite the importance of heme for these two bacterial pathogens, to our knowledge, the effect of heme utilization on the interplay between these microbes and iron-sequestering host-defense proteins has received little attention.

Our current results indicate that the use of heme as an iron source protects *S. aureus* from iron withholding by CP (Fig. S8), suggesting that heme may be preferentially utilized over Fe(II) and Fe(III) in the presence of CP. Prior work demonstrated that when both transferrin and heme (or hemoglobin) are provided to *S. aureus* in culture, it will preferentially use heme (16, 50). Moreover, in the presence of heme, *S. aureus* can delay siderophore biosynthesis via the heme-responsive regulator SbnI, suggesting that it will prioritize heme uptake over siderophore-mediated iron acquisition (51). These earlier studies exemplify the ability of bacterial pathogens to adapt their iron uptake strategies in response to certain host-defense factors. Our current work indicates a similar response to CP, where *S. aureus* and *P. aeruginosa* respond to iron withholding by CP by utilizing heme to meet metabolic iron requirements. These observations underscore the importance of pathogen adaptation to the presence of host-defense factors, which undoubtedly vary in abundance over an infection time course.

Other factors, including local oxygen tension, can affect how bacteria prioritize one iron source over others. The availability of Fe(II) and Fe(III) is highly variable based on oxygen levels and redox environment. At infection sites that are considered microaerobic or anaerobic, pathogens may depend more on uptake of Fe(II) or heme over Fe(III). In addition, it is possible that temporal variation of oxygen levels may lead to the primary utilization of one iron source over others. Infections of *P. aeruginosa* in the CF lung have provided a useful model for understanding how reduced oxygen availability affords changes in iron-acquisition strategies, and it is likely that the findings from this model apply to other infections. As *P. aeruginosa* infection of the CF lung progresses, oxygen availability decreases over time because of decreased lung function and the formation of thick biofilms (52, 53). The evolution of iron-acquisition strategies used by *P. aeruginosa* in the CF lung reflects these physical determinants of iron availability. Iron acquisition via siderophore systems is prioritized in acute infection, but reduction of siderophore production and even evolution to non–pyoverdine-producing strains as infection progresses indicates a reduced dependence of Fe(III) acquisition (8, 14, 28). In contrast, late-stage *P. aeruginosa* isolates of CF lung infection prioritize heme utilization to satisfy their iron needs (13, 14, 28). In addition to reduced oxygen availability over time, this shift to heme utilization is expected based on the damage to surrounding tissues that accumulates over time and causes capillary leakage into the infection site, providing access to heme (54). Prior work has shown that CP accumulates as CF lung infection progresses and its levels correlate with disease severity (55, 56). Based on our current findings, it is intriguing to consider that iron limitation by CP contributes to *P. aeruginosa* shifting toward a preference for heme utilization *in vivo*. More broadly, the possibility that heme utilization is particularly important for diverse bacterial pathogens at chronic inflammatory sites where CP is abundant warrants investigation.

Our prior work showed that iron limitation, but not zinc or manganese limitation, afforded significant growth inhibition of *P. aeruginosa* (20). Guided by this observation, we reason that the observed antibacterial activity of CP against *P. aeruginosa* is primarily attributable to iron limitation under these conditions. The impact of heme on the growth-inhibitory activity of CP is in agreement with these prior studies; heme utilization allows for sufficient iron acquisition in the presence of CP, which lessens the impact of CP-mediated iron withholding on *P. aeruginosa* growth. By contrast, our growth-inhibitory activity study suggests an alternative scenario for *S. aureus*. Because heme is toxic to *S. aureus*, the pathogen may be forced to balance heme toxicity with the requirement for iron when extracellular non-heme iron is limited by CP. Our current results suggest less heme toxicity in CP-treated cultures of *S. aureus*, which may be attributable to the growth promotion expected from recovering cellular iron levels. Thus, it is possible that heme utilization protects *S. aureus* from the growth-inhibitory activity of CP, but this possibility requires further evaluation because of the competing effects of heme toxicity. Regardless, the cellular metal inventory of *S. aureus* indicates that it is a less Fe-centric organism than *P. aeruginosa* (57), and various experimental observations indicate that the growth-inhibitory activity of CP against *S. aureus* is not solely attributable to sequestration of iron. For instance, our prior metal-supplementation studies indicated that limitation of manganese, iron, and zinc can contribute to the growth-inhibitory activity of CP against *S. aureus* (20). Furthermore, the effect of manganese withholding by CP on *S. aureus* growth and virulence has been extensively studied and has revealed a substantial role for manganese withholding by CP in host defense during *S. aureus* infections (24, 34, 35, 36, 37, 38).

Our growing understanding of the Fe(II)-sequestering properties of CP suggests that it may affect iron homeostasis in a number of bacterial pathogens during infection. However, our current results indicate that for bacterial infections where heme is available as an iron source, CP may be less capable of eliciting iron-starvation responses by the pathogen. Changes in bacterial iron homeostasis have not been reported in murine models of infection using CP-KO mice (24, 26, 45, 58, 59). However, it is worthwhile to consider that heme utilization may have mitigated responses to iron withholding by CP *in vivo*. For *P. aeruginosa* and *S. aureus* in particular, the current work suggests that heme utilization may prevent iron starvation by CP during infection and may consequently alter their dynamics during coinfection. A recent study indicated that heme levels in the CF lung negatively correlate with lung function (60). Thus, the possibility that heme enables resistance of *S. aureus* and *P. aeruginosa* to iron-sequestering host factors such as lactoferrin and CP in the CF lung, and ultimately contributes to loss of lung function, warrants further investigation. In closing, these studies expand our understanding of the competition for iron between CP and two prominent bacterial pathogens, *P. aeruginosa* and *S. aureus*, and provide a new perspective for the complex role of this innate immune protein in iron homeostasis during infection.

## Experimental procedures

### General materials and methods

#### Solutions and buffers

All chemicals were acquired from commercial suppliers and used as received. All solutions were prepared using Milli-Q water (18.2 MΩ cm). All buffer solutions were filtered (0.2 μm) before use. Stock solutions of metal ions (CaCl_2_ [1.0 M], MnCl_2_·4H_2_O [100 mM], (NH_4_)_2_Fe(SO_4_)_2_·6H_2_O [100 mM], NiSO_4_·6H_2_O [100 mM], CuSO_4_·5H_2_O [100 mM], ZnCl_2_ [1.0 M] [Sigma]) were prepared in Milli-Q water in acid-washed volumetric glassware and transferred into polypropylene containers. For stock solutions of Fe(II), (NH_4_)_2_Fe(SO_4_)_2_·6H_2_O powder was transferred into a glove box, dissolved with Milli-Q water, and stored in the glove box. Ultrapure Tris (VWR) was used to prepare Tris buffers and the Tris:TSB medium.

#### General CP methods

Both CP and CP-Ser [S100A8(C42S)/S100A9(C3S) oligomer] were used in this work. CP-Ser has been used routinely for biochemical, biophysical, and functional studies of CP and exhibits comparable metal binding properties and antimicrobial activity to native CP (25). The CP heterodimer was prepared and stored as described previously (25). Biotinylated CP was prepared as described previously (29). Protein aliquots were thawed only once, immediately before use. If stored in the standard purification buffer (20 mM HEPES, 100 mM NaCl, pH 8.0, for CP-Ser; for CP, the buffer also contained 5-mM DTT), the protein was buffer-exchanged three times into 20-mM Tris and 100 mM NaCl, pH 7.5, using presterilized 0.5-mL 10K molecular weight cut-off spin concentrators (Amicon) before being used. Protein concentrations are reported for the CP heterodimer and were determined by A_280_ using the calculated extinction coefficient of the CP heterodimer (ε_280_ = 18,450 M^−1^ cm^−1^) obtained from the online ExPASy ProtParam tool.

### Instrumentation

#### Optical absorption spectroscopy

Optical absorption spectra and absorbance at 600 nm were recorded on a Beckman Coulter DU-800 spectrophotometer operated at an ambient temperature. Disposable plastic cuvettes (1-cm path length) were used to measure absorbance at 600 nm.

#### HPLC

HPLC was performed on an Agilent 1200 instrument equipped with a thermostatted autosampler set at 4 °C, a thermostatted column compartment set at 20 °C, a multiwavelength detector set at 220, 280, and 365 nm (500-nm reference wavelength, 40-nm bandwidth), and a fluorescence detector set at λ_ex_ = 398 nm and λ_em_ = 455 nm for pyoverdine detection. For all HPLC runs, solvent A was 0.1% TFA in water and solvent B was 0.1% TFA in acetonitrile. A Clipeus C18 column (120 Å, 4.6 × 250 mm, Higgins Analytical, Inc) and a flow rate of 1 ml/min were used for all analytical HPLC. Elution gradients are given for specific experiments below.

#### Microwave digestion

For metal analysis of bacterial suspensions, bacterial suspensions were liquefied using a Milestone UltraWAVE digestion system housed in the Center for Environmental Health Sciences Core Facility at the Massachusetts Institute of Technology (MIT). A standard microwave protocol (15-min ramp to 180 °C at 1500 W power; 10-min ramp to 220 °C at 1500 W power) was used for the acid digestion.

#### ICP–MS

Metal-ion concentrations were quantified by using an Agilent 7900 inductively coupled plasma–mass spectrometer housed in the Center for Environmental Health Sciences Bioanalytical Core Facility at MIT. The instrument was operated in the helium mode. The instrument was calibrated before each analysis session using a series of five serially diluted (1:10) samples of the Environmental Calibration Standard (Agilent, part # 5183-4688) in 5% nitric acid and a 5% nitric acid–only standard. The concentrations of Mg, Ca, Mn, Fe, Co, Ni, Cu, and Zn were quantified, and terbium (1 ppb Tb; Agilent, part # 5190-8590) was used as an internal standard. Samples were prepared in 15-mL Falcon tubes, and 2-mL samples were transferred to ICP–MS polypropylene vials (Perkin Elmer, B3001566) and analyzed.

### General microbiology methods

Strains and plasmids used in this study are listed in Table S2.

#### Bacterial growth media

The TSB (VWR) and LB media (BD Life Sciences) were prepared as recommended by the supplier. The TSB was supplemented with 0.25% dextrose from a sterile solution of 40% dextrose (Sigma). Tris:TSB is a 62:38 mixture of 20 mM Tris, 100 mM NaCl, pH 7.5, and TSB. This medium was supplemented with 2 mM Ca(II) from a 1 M stock in Milli-Q water and sterilized by filtration (0.2 μm). ICP-MS analyses of the Tris:TSB media and LB media are reported in Table S1.

#### Preparation of CDM

Metal-depleted CDM was prepared as described previously (27). This medium was supplemented with 1 mM Ca(II), 0.3 μM Mn(II), 5 μM Fe(II), 0.1 μM Ni(II), 0.1 μM Cu(II), and 6 μM Zn(II).

#### Heme stock preparation

For assays involving heme, a fresh stock from hemin was prepared within 30 min before the assay and the stock concentration was measured using a modified pyridine hemochrome assay (61). Hemin (50 mg, Sigma) was washed with 100 mM HCl (1 ml). The hemin was dissolved in 100 mM NaOH in 20 mM Tris (10 ml), at which point, the solution turned dark green. The suspension was adjusted to a pH of 7.0 using 100 mM HCl and sterilized by filtration through a 0.2-μm filter. To measure the concentration of the prepared heme stock, a 1:500 dilution was prepared into 1 ml of 20 mM Tris, pH 8.0, in a quartz cuvette. Pyridine (100 μl, Sigma) and sodium hydrosulfite (50 g, Sigma) were added to the cuvette and mixed, and the suspension turned light pink. Absorbance at 418, 525, and 555 nm were measured. The heme concentration was determined using the calculated extinction coefficients for pyridine hemochrome (170 mM^−1^ cm^−1^ at 418 nm, 17.5 mM^−1^ cm^−1^ at 525 nm, and 34.5 mM^−1^ cm^−1^ at 555 nm).

### Metal-uptake assay

Bacterial strains listed in Table S2 were streaked from freezer stocks onto the TSB (*Pa*) or LB (*Sa*) 1.5% agarose plates and grown overnight (12–16 h) at 37 °C. A single colony was used to inoculate 2-ml TSB (*Pa*) or LB (*Sa*) and incubated overnight (12–16 h) on a rotating wheel at 37 °C. The overnight culture was diluted 1:100 into 2 ml Tris:TSB + 2 mM Ca(II), CDM + 1 mM Ca(II), or LB + 2 mM Ca(II). Heme-treated cultures were supplemented with 5 μM heme. Cultures were supplemented with 10 μM (*Pa*) or 20 μM (Sa) CP or CP-Ser. Diluted cultures were incubated for 8 h at 250 rpm, 37 °C. The optical density (absorbance at 600 nm) of each culture was measured, and the cells were harvested by centrifugation (3750 rpm, 4 °C, 5 min). Supernatants (1 ml for each culture) were stored in a −20 °C freezer until further use in the CAS assay or HPLC analysis. Cell pellets were washed by a three-step procedure first with 1 ml Tris buffer (20 mM Tris, 100 mM NaCl, pH 7.5), second with 1 ml Tris buffer + 500 μM EDTA (VWR, Product M101-500G), and third with 1 ml Tris buffer. Cells were resuspended in Tris buffer to an absorbance at 600 nm of 10, and 200 μl of the suspension was diluted with 1.8 ml of 5% nitric acid. The resulting samples were liquefied by microwave digestion and analyzed by ICP–MS. Metal concentrations correspond to the metal content of an absorbance at 600 nm = 10 cell suspensions. The mean metal content, SEM, and *p* values from a two-tailed *t*-test assuming unequal variances are reported. This assay often results in data with large SDs because of the nature of day-to-day differences in culturing. For this reason, we perform a minimum of five replicates for each condition.

### CAS assay

A CAS assay solution was prepared according to a previously published procedure (40). Briefly, hexadecyltrimethylammonium bromide (21.9 mg, Spectrum Chemical) was dissolved in 25 ml Milli-Q water while stirring. FeCl_3_·6H_2_O (1.5 ml, 1 mM, Sigma) dissolved in 10 mM HCl was mixed with CAS (2 mM, 7.5 ml, MP Biomedicals) and slowly added to the hexadecyltrimethylammonium bromide solution. The resultant solution was transferred to a volumetric flask. 2-Ethanesulfonic acid (MES, 9.76 g, Sigma) was dissolved in 50-ml Milli-Q water, and the pH was adjusted to 5.6. This buffer solution was added to the volumetric flask, and the volume was adjusted to 100 ml before filtering the solution through a 0.2-μm filter. This solution was stored at 4 °C until use. Immediately before use, 5 mM sulfosalicylic acid (MP Biomedicals) was added to make a working solution. Supernatants harvested from metal-uptake assay cultures were used for the CAS assay analysis of siderophore levels. For cultures treated with biotinylated CP, 75 μl of streptavidin agarose resin (Pierce, Thermo Scientific) was washed 3× with Tris buffer (20 mM Tris, 100 mM NaCl, pH 7.5) before adding 600 μl culture supernatant to the resin. Samples were incubated at RT for 45 min before pelleting the resin by centrifugation (13,000 rpm, 1 min) and removing 500 μl of the supernatant. The supernatant (500 μl) was added to the CAS assay solution (500 μl) and incubated in the dark at RT for 45 min. Samples were transferred to cuvettes and A_630_ was measured. A_630_ measurements were normalized to absorbance at 600 nm of the culture corresponding to each supernatant sample to normalize for cell density. Three biological replicates, mean A_630_/absorbance at 600 nm, and *p* values from a two-tailed *t*-test assuming unequal variances are reported.

### ^57^Fe-uptake assay

#### Stock preparation

^57^Fe (5 mg) was dissolved in 1.75 ml of 100 mM H_2_SO_4_ in an anaerobic glove box to make a 50 mM solution of ^57^FeSO_4_. This solution was incubated in the glove box at 65 °C for 20 h to aid dissolution. After incubation, the solution was vortexed and heated at 80 °C for 10 min. After this incubation, the ^57^Fe was completely dissolved. This stock was diluted to 500 μM in Milli-Q water, and its concentration was measured using a ferrozine assay, as described previously (62).

#### Assay setup

*P. aeruginosa* PA14 was streaked from freezer stocks onto a TSB 1.5% agarose plate and grown overnight (12–16 h) at 37 °C. A single colony was used to inoculate 2 ml TSB and incubated overnight (12–16 h) on a rotating wheel at 37 °C. The overnight culture was diluted 1:100 into 2 ml CDM (prepared without Fe(II)). Heme-treated cultures were supplemented with 5 μM heme stock solution. CP-treated cultures were supplemented with 10 μM CP. Cultures were grown, washed, and analyzed as described in the Metal-uptake assay section.

### RT-PCR

*P. aeruginosa* PAO1 and PA14 was streaked on TSB 1.5% agarose plates and incubated at 37 °C for 16 h. A single colony was used to inoculate 3 ml TSB, and the culture was incubated at 37 °C for 16 h at 250 rpm. This culture was diluted 1:100 into 5 mL CDM in an acid-washed 50-mL flask and supplemented with CP-Ser (10 μM) or an equivalent volume of buffer (20 mM Tris, 100 mM NaCl, pH 7.5). This diluted culture was grown at 37 °C for 16 h at 250 rpm, and an aliquot of culture (500 μl) was combined with RNAlater (Sigma, 500 μl) before RNA extraction. RNA was extracted using an RNeasy minikit (Qiagen) according to the manufacturer's directions. RNA (50 ng/μl) was used to generate cDNA with an ImProm-II cDNA synthesis kit (Promega). A StepOnePlus instrument (Applied Biosystems) and TaqMan reagents (Life Technologies) were used to analyze cDNA. Primers and probes for *hasA*, *hasR*, *phuS*, and *oprF* are listed in Table S3. Relative RNA levels were normalized to the levels of the *oprF* mRNA, and fold changes in mRNA relative to the untreated control are shown. Both individual data points from three biological replicates and the mean fold change are reported with *p* values from a two-tailed *t*-test assuming unequal variances.

*S. aureus* USA300 JE2 was streaked on LB 1.5% agarose plates and incubated at 37 °C for 16 h. A single colony was used to inoculate 3 ml LB, and the culture was incubated at 37 °C for 16 h on a rotating wheel. This culture was diluted 1:100 into 2.5 ml LB + 2 mM Ca(II) in a plastic culture tube and supplemented with CP (20 μM) or an equivalent volume of buffer (20 mM Tris, 100 mM NaCl, pH 7.5). This diluted culture was grown at 37 °C for 8 h at 250 rpm. Cells were harvested from an aliquot of culture (2 ml) and resuspended in 100 μl RNase-free water supplemented with 2.5 μg/ml lysostaphin (Sigma) and 0.25 μg/ml lysozyme (Sigma). Cell suspensions were incubated for 15 min at 37 °C before being extracted using an RNeasy minikit (Qiagen) according to the manufacturer's directions. RNA (50 ng/μl) was used to generate cDNA with an ImProm-II cDNA synthesis kit (Promega). A LightCycler 480 II Real-Time PCR instrument and SYBR reagents (Bio-Rad) were used to analyze cDNA. Primers for *sigA*, *sirA*, and *isdC* are listed in Table S3. Relative RNA levels were normalized to the levels of the *sigA* mRNA and fold changes in mRNA relative to the untreated control are shown. Both individual data points from three biological replicates and the mean fold change are reported with *p* values from a two-tailed *t*-test assuming unequal variances.

### Growth-curve assay

*P. aeruginosa* PAO1, *P. aeruginosa* PA14, *S. aureus* USA300 JE2, and *S. aureus* USA300 Newman were streaked from freezer stocks onto TSB (*Pa*) or LB (*Sa*) 1.5% agar plates and grown overnight at 37 °C. A single colony was used to inoculate 2 ml of TSB (*Pa*) or LB (*Sa*) and grown overnight at 37 °C on a rotating wheel. Overnight cultures were diluted 1:100 into 2-ml Tris:TSB (*Pa*) or LB (*Sa*) and incubated at 37 °C on a rotating wheel until cultures reached absorbance at 600 nm ≈ 0.6. These mid-log phase cultures were diluted 1:500 into Tris:TSB (Pa) or LB (*Sa*) supplemented with 2 mM Ca(II) and 0, 2.5, 5, or 10 μM heme. CP was buffer-exchanged into the sterile Tris buffer (20 mM Tris, 100 mM NaCl, pH 7.5), and 10× protein stocks were prepared in Tris buffer. To each well in a 96-well plate, 10 μl of 10× CP stock and 90 μl of diluted culture were added. Each condition was performed in technical triplicate, and three biological replicates were included. Plates were tightly wrapped with a moist paper towel and plastic wrap to prevent evaporation and incubated at 37 °C, 150 rpm. Absorbance at 600 nm values were measured at 0, 2, 4, 6, 8, 10, 12, 16, 20, and 24 h using a BioTek Synergy HT plate reader. The mean absorbance at 600 nm values and SEM are reported.

### β-Galactosidase assay

A β-galactosidase assay was used to determine the effect of heme on *antR* translation by *P. aeruginosa*. *P. aeruginosa* PAO1/P_*antR*_-‘*lacZ*^*-*SD^ was streaked from a freezer stock onto a 1.5% TSB agar plate and grown overnight (12–16 h) at 37 °C. Three colonies were used to inoculate TSB (5 ml) and incubated overnight (12–16 h) on a rotating wheel at 37 °C. The overnight cultures were diluted 1:100 into 2 ml Tris:TSB with 2 mM Ca(II). CP-treated samples were supplemented with 10 μM CP, and heme-treated samples were supplemented with 5 μM heme. Diluted cultures were incubated for 8 h at 37 °C and 250 rpm. The optical density (absorbance at 600 nm) of each culture was measured, and 1 ml of each cell sample was harvested by centrifugation (13,000 rpm, 5 min). Cell pellets were resuspended in 1 ml potassium phosphate buffer (50 mM, pH 7.0), and 100 μl of each resuspension was diluted into 900 μl Z-buffer (60 mM Na_2_HPO_4_, 35 mM NaH_2_PO_4_, 1 mM KCl, 100 mM MgSO_4_, 50 mM β-mercaptoethanol [all components acquired from Sigma]). Cells were lysed by adding chloroform (100 μl) and 0.1% SDS (50 μl), and samples were vortexed until they turned cloudy white. The reaction was initiated with the addition of *o*-nitrophenyl-β-D-galactopyranoside (200 μl of a 4 mg/ml stock, Thermo). Samples were vortexed and incubated at RT for 20 min. The reaction was quenched using sodium carbonate (1 M). The quenched reaction solution was centrifuged at 13,000*g* to remove cell debris and the absorbance (A_420_) of the supernatant was determined. β-Galactosidase activity was quantified as Miller units calculated using the following equation: (1000 × A_420_)/(time [min] × volume [mL] × A_600nm_). Both individual data points and the mean Miller units are reported with *p* values from a two-tailed *t*-test assuming unequal variances.

### Metabolite analysis by HPLC

HPLC analysis was used to quantify the effect of CP and heme on pyoverdine and phenazine production by *P. aeruginosa* PA14. Supernatants from cultures used in the metal-uptake assay were thawed at RT and centrifuged at 13,000 rpm for 5 min. For pyoverdine analysis, the method used was 0 to 35% B over 30 min, 1 ml/min, and a 10-μl sample of the supernatant or sample was injected. Pyoverdine standard was prepared as described previously (22). A single representative biological replicate is reported. The method for detecting phenazines was 15 to 80% B over 20 min, 1 ml/min with a 50-μl sample of the supernatant or sample being injected. For phenazine quantification, integration at 365 nm was converted to the concentration using a standard curve of PYO or PCA (500, 100, 50, 10, 5, 2.5, and 1.25 μM). The calculated phenazine concentrations were normalized to culture absorbance at 600 nm. For phenazine quantification, data from three biological replicates, mean normalized concentrations, and *p* values from a two-tailed *t*-test assuming unequal variances are reported.

## Data availability

All data are presented in the main text and Supporting information. Original data files, plasmids, strains, and protein samples (when a laboratory is unable to do protein purification) will be made available upon request.

## Conflict of interest

The authors declare that they have no conflicts of interest with the contents of this article.
