# Supplementary material for: Heme protects Pseudomonas aeruginosa and Staphylococcus aureus from calprotectin-induced iron starvation
Source: J Biol Chem. 2020 Dec 9;296:100160. doi: 10.1074/jbc.RA120.015975 (PMC7948498; doi:10.1074/jbc.RA120.015975)
Supplement: Supplementary Material [file mmc1.pdf]

Heme protects *Pseudomonas aeruginosa* and *Staphylococcus aureus* from calprotectin-induced iron starvation

Emily M. Zygiel[1], Adunoluwa O. Obisesan[1], Cassandra E. Nelson[2], Amanda G. Oglesby[3,*], Elizabeth M. Nolan[1,*]

[1] Department of Chemistry, Massachusetts Institute of Technology, Cambridge, MA 02139, USA

[2] School of Pharmacy, Department of Pharmaceutical Sciences, University of Maryland, Baltimore, MD 21201, USA

[3] School of Medicine, Department of Microbiology and Immunology, University of Maryland, Baltimore, MD, 21021, USA

[*]Corresponding author:

lnolan@mit.edu, Phone: 617-452-2495

aoglesby@rx.umaryland.edu, Phone: 410-706-8650

TABLE OF CONTENTS

Supplementary tables

Table S1. ICP-MS analysis of LB and Tris:TSB media………………………………………………. S3

Table S2. Strains used in this study……………….……………………………….......................... S4

Table S3. Primers and probes used for RT-PCR…..……………………………………………..,… S5

Table S4. OD_{600} values of *S. aureus* cultures following CP and heme treatment……………………… S6

Supplementary figures

Figure S1. Effect of CP and heme on PAO1 metal uptake ………………………….……................. S7

Figure S2. Effect of CP and heme on PA14 metal uptake in CDM…………………………………… S8

Figure S3. Effect of CP and heme on PA14 metal uptake in Tris:TSB……………………………….. S8

Figure S4. CP inhibits metal uptake by multiple *S. aureus* strains. ..……………………………….. S9

Figure S5. CP does not affect the CAS assay**.**……………………...………………………………..... S10

Figure S6. Effect of CP and heme on *S. aureus* metal uptake………………...……………………..… S11

Figure S7. Effect of CP and heme on the growth of *P. aeruginosa* and *S. aureus* …………………..... S12

Figure S8. Model for the effect of heme on CP-mediated iron starvation in *S. aureus* based on the current work…………………………………………………………………………………….. S13

Supporting References………………………………………………………………………....... S14

Table S1. Representative ICP-MS analysis of LB and Tris:TSB media.

Element	LB (μM)	Tris:TSB (μM)
**Mg**	77.8	164.0
**Ca**	149	79.1
**Mn**	0.275	0.157
**Fe**	7.89	4.25
**Co**	0.164	0.052
**Ni**	0.001	1.14
**Cu**	0.214	0.257
**Zn**	15.6	5.39

Table S2. Strains used in this study.

Strain	Description	Reference
S. aureus USA300	Methicillin-resistant strain	(1)
S. aureus USA300 JE2	Parent strain of NTML Collection; community isolate; methicillin-resistant	(2)
S. aureus Newman	Laboratory strain	(3), (4) Courtesy of Tony Richardson
S. aureus P1	Longitudinal CF isolate, type USA300, #2050	(5)
S. aureus P2	Longitudinal CF isolate, type USA300, #2059	(5)
S. aureus P3	Longitudinal CF isolate, type USA300, #2089	(5)
S. aureus COL	Methicillin-resistant strain	MRSA Clinical Isolate, 1961, Courtesy of Tony Richardson
S. aureus M2	Laboratory strain of MRSA	(6)
P. aeruginosa PAO1	*Pa* laboratory strain	(7)
P. aeruginosa PAO1 Δ*hasRphuR*	*hasR and phuR* knockout strain	(8)
P. aeruginosa PA14	Clinical isolate UCBPP-PA14	(9), Courtesy of Dianne Newman
P. aeruginosa PAO1/P_{antR}-'$lacZ^{-SD}$	PAO1 with the P_{antR}-'$lacZ^{-SD}$ reporter fusion integrated at the chromosomal *att* site	(10)

Table S3. Primers and probes used for RT-PCR.

Target	Sequence	Reference
Primers		
oprf (f)	GCG TTC GCA ACA TGA AGA AC	(11)
oprf (r)	CTT CTT GTT GCC GGT TTC GTA	(11)
phuS (f)	TGC CGA CGA ACA CCA TGA	(12)
phuS (r)	TGG CGA CCT GGC GAA A	(12)
hasA (f)	ATC GAC GCG CTG CTG AA	(13)
hasA (r)	TGG TCG AAG GTG GAG TTG ATC	(13)
hasR (f)	CGT GGC GTC GAG TAC CAG	(13)
hasR (r)	GGT CTT CGA ACA GAA GTC GTT G	(13)
sigA (f)	TGG TGC TGG ATC TCG ACC TA	
sigA (r)	TGC AAT TGC TGA CCA AGC AC	
sirA (f)	ACG CGA CAA TTA AGT CCG GT	
sirA (r)	CCA CTG ACG TCG CTG TAT CT	
isdC (f)	CTG CCA AAG ATG AAC GCA CT	
isdC (r)	GCA CCT GCT ACA TCA GTT GGT	
Probes		
oprf	CGG TGA GTA CCA TGA CGT TCG TGG C	(11)
hasA	TCG ACC CGA GCC TGT	(13)
hasR	CTG GCC TAC GGG CAG CTC TCC TA	(13)
phuS	CTT TCG GCC GCC GCT TCG A	(12)

Table S4. OD_{600} values of *S. aureus* cultures following CP and heme treatment.

Treatment	JE2 OD_{600} (range, N = 5)
**Untreated**	3.7 – 3.9
**CP**	2.3 – 3.1
**Heme**	3.2 – 3.8
**CP + heme**	2.3 – 2.5

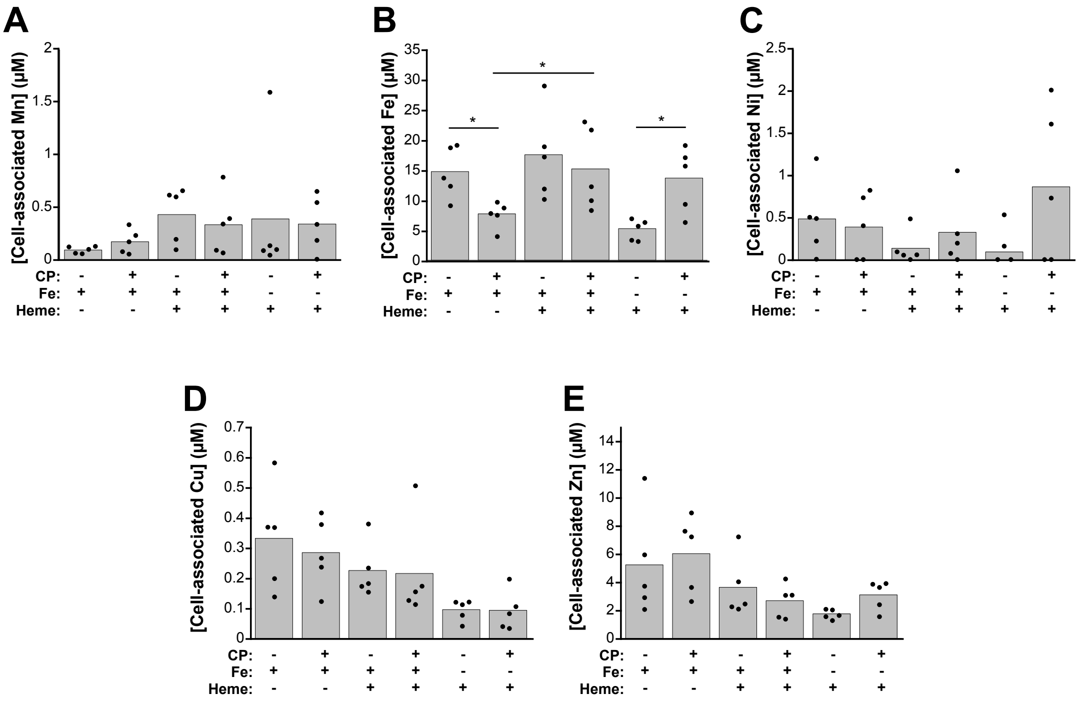

Figure S1. Effect of CP and heme on PAO1 metal uptake. Cell-associated metal in *P. aeruginosa* PAO1 grown in CDM or Fe-depleted CDM in the absence or presence of CP (10 µM) and heme (5 µM) (N = 5, *$P < 0.05$). CP, calprotectin; CDM, chemically defined medium.

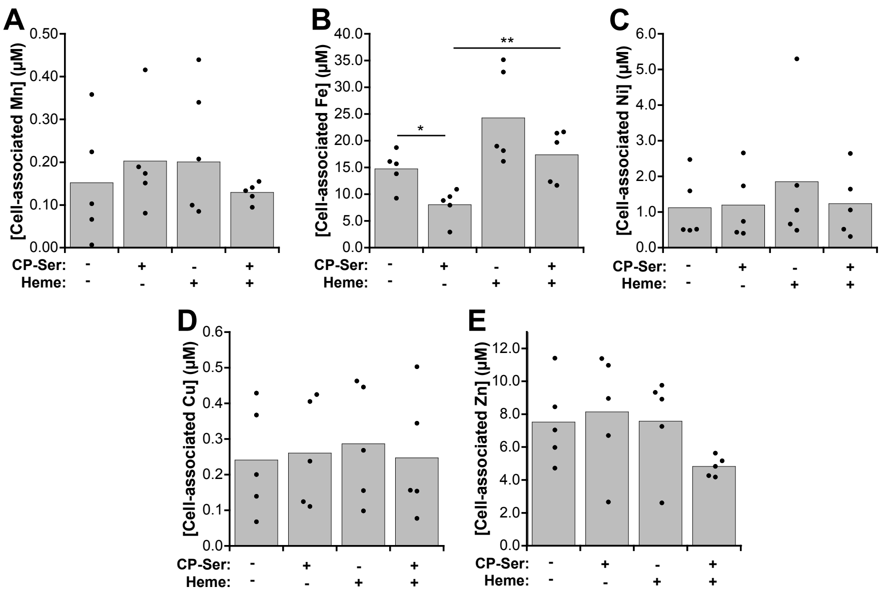

Figure S2. Effect of CP and heme on PA14 metal uptake in CDM. Cell-associated metal in *P. aeruginosa* PA14 grown in CDM in the absence or presence of CP-Ser (10 µM) and heme (5 µM) (N = 5, **P* < 0.05, ** *P* < 0.01). CP, calprotectin; CDM, chemically defined medium.

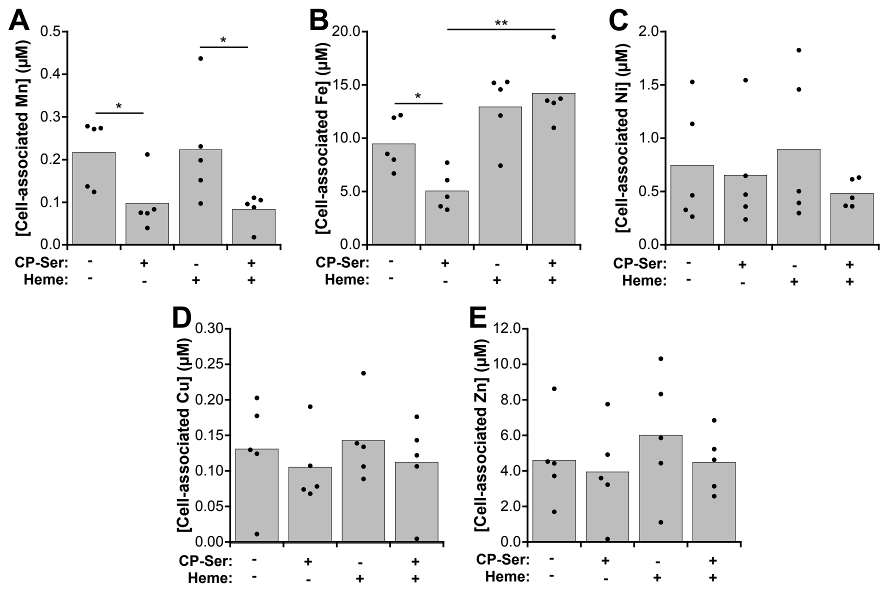

Figure S3. Effect of CP and heme on PA14 metal uptake in Tris:TSB. Cell-associated metal in *P. aeruginosa* PA14 grown in Tris:TSB in the absence or presence of CP-Ser (10 µM) and heme (5 µM) (N = 5, **P* < 0.05, ** *P* < 0.01). CP, calprotectin.

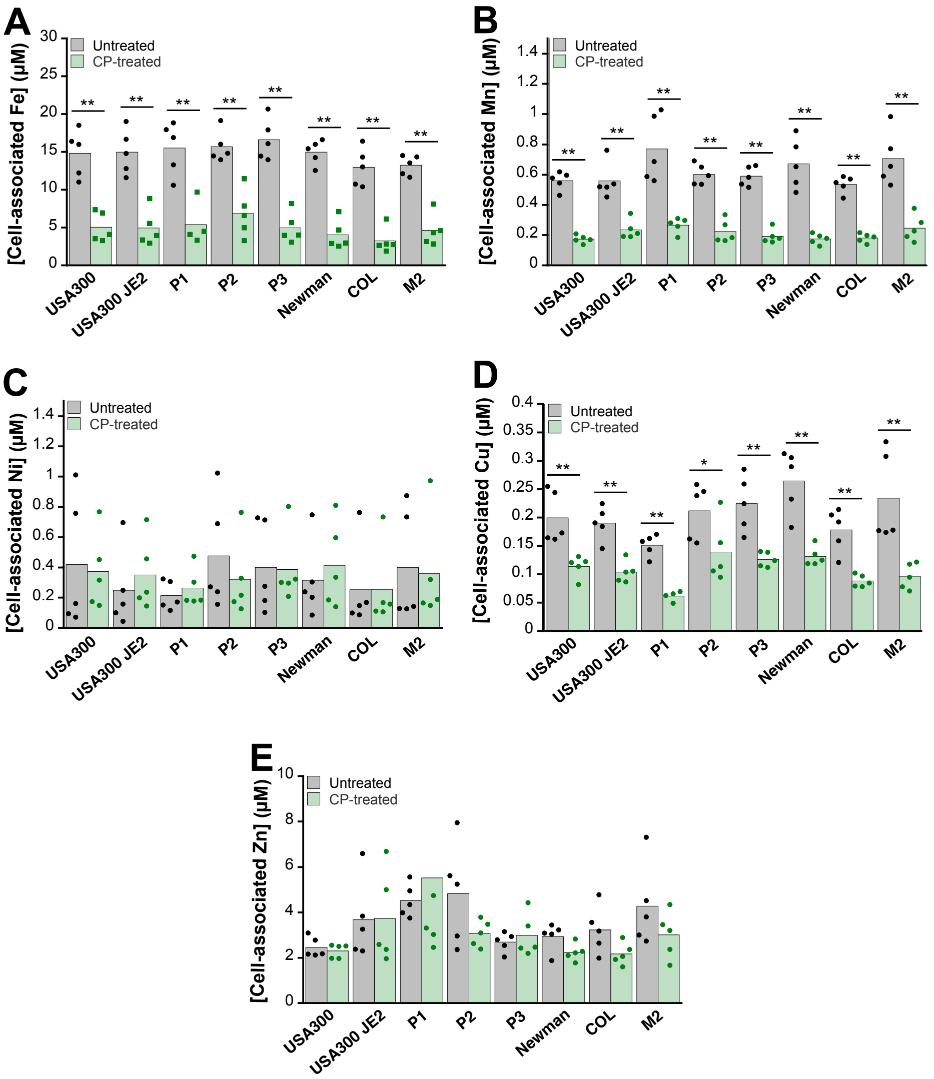

Figure S4. CP inhibits metal uptake by multiple *S. aureus* strains. Cell-associated metal levels for *S. aureus* strains (Table S2) grown in LB in the absence or presence of CP (20 μM) (N = 5, **P* < 0.05, ***P* < 0.01). CP, calprotectin.

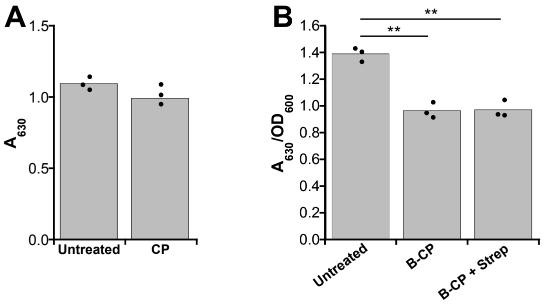

Figure S5. CP does not affect the CAS assay. (A) CAS dye (500 µL) was incubated with 500 µL Tris buffer (untreated) or 500 µL 20 µM CP prepared in Tris buffer (CP) for one hour before measuring the absorbance at 630 nm. (B) *S. aureus* USA300 JE2 was grown in LB the absence or presence of B-CP (20 µM). Supernatants harvested from B-CP treated cultures were split into two separate aliquots and B-CP was pulled down via treatment with streptavidin agarose resin (+ Strep) in one aliquot while the other was left untreated. A CAS assay was performed on each supernatant aliquot. A_{630} measurements were normalized to culture OD_{600} (N = 3, $^{**}P < 0.01$). CP, calprotectin.

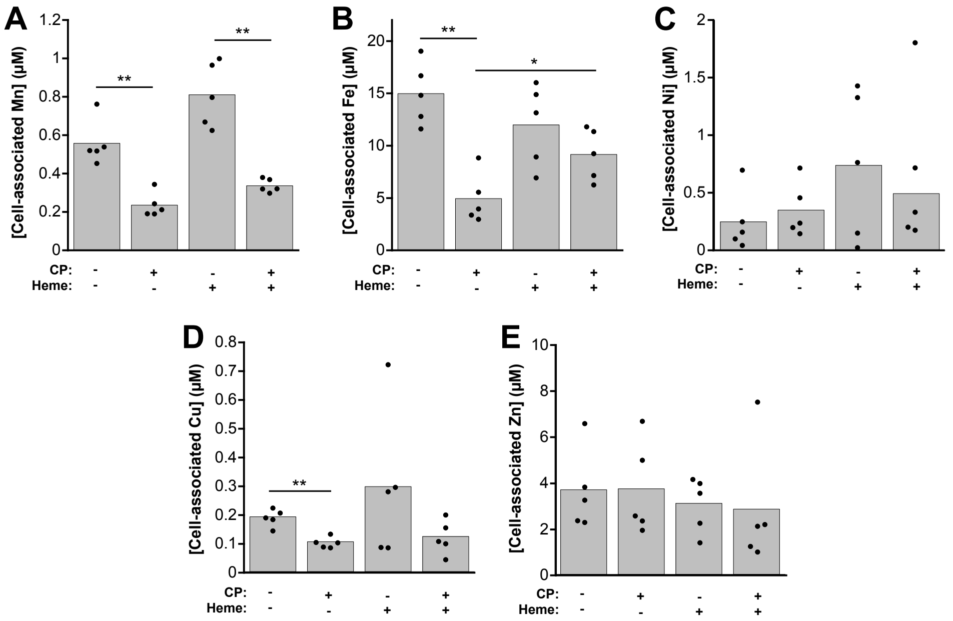

Figure S6. Effect of CP and heme on *S. aureus* metal uptake. Cell-associated metal for *S. aureus* USA300 JE2 grown in LB in the absence or presence of CP (20 μM) and heme (5 μM). (N = 5, **P* < 0.05, ***P* < 0.01). CP, calprotectin.

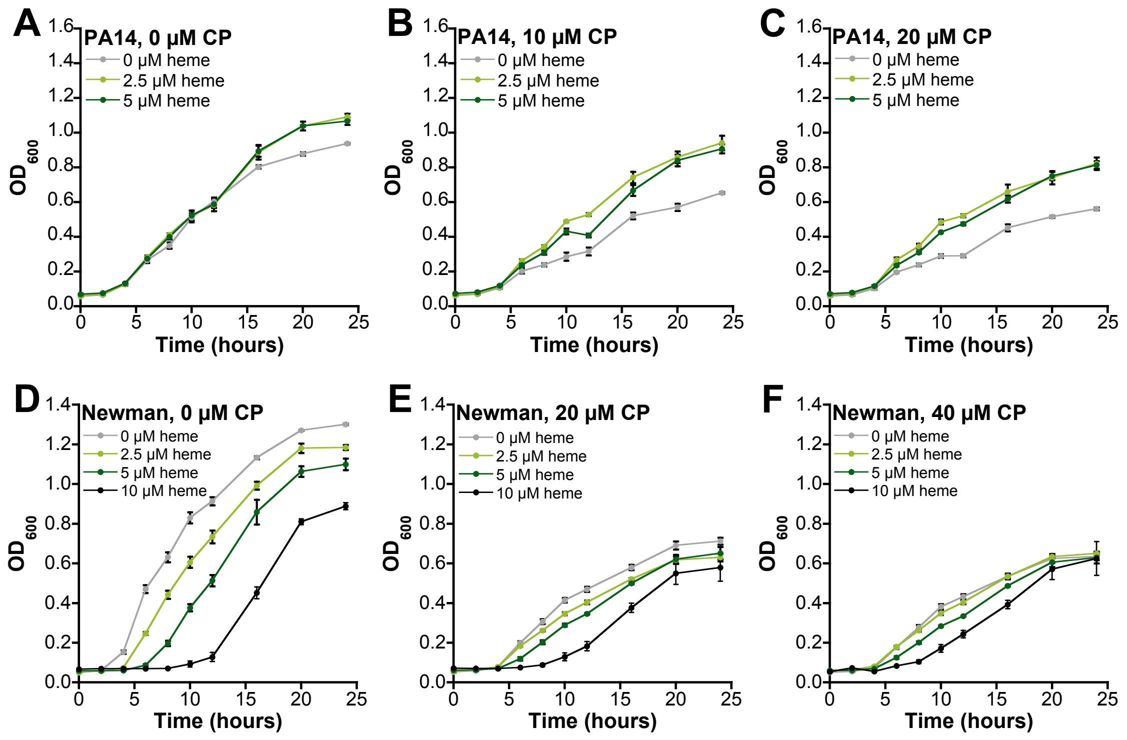

Figure S7. Effect of CP and heme on the growth of *P. aeruginosa* and *S. aureus*. (A-C) *P. aeruginosa* PA14 was grown in Tris:TSB and treated with 0, 10, or 20 µM CP and 0, 2.5, or 5 µM heme. (D-F) *S. aureus* Newman was grown in LB with 0, 20, or 40 µM CP and 0, 2.5, 5, or 10 µM heme (N = 3, SEM, **P* < 0.05). CP, calprotectin.

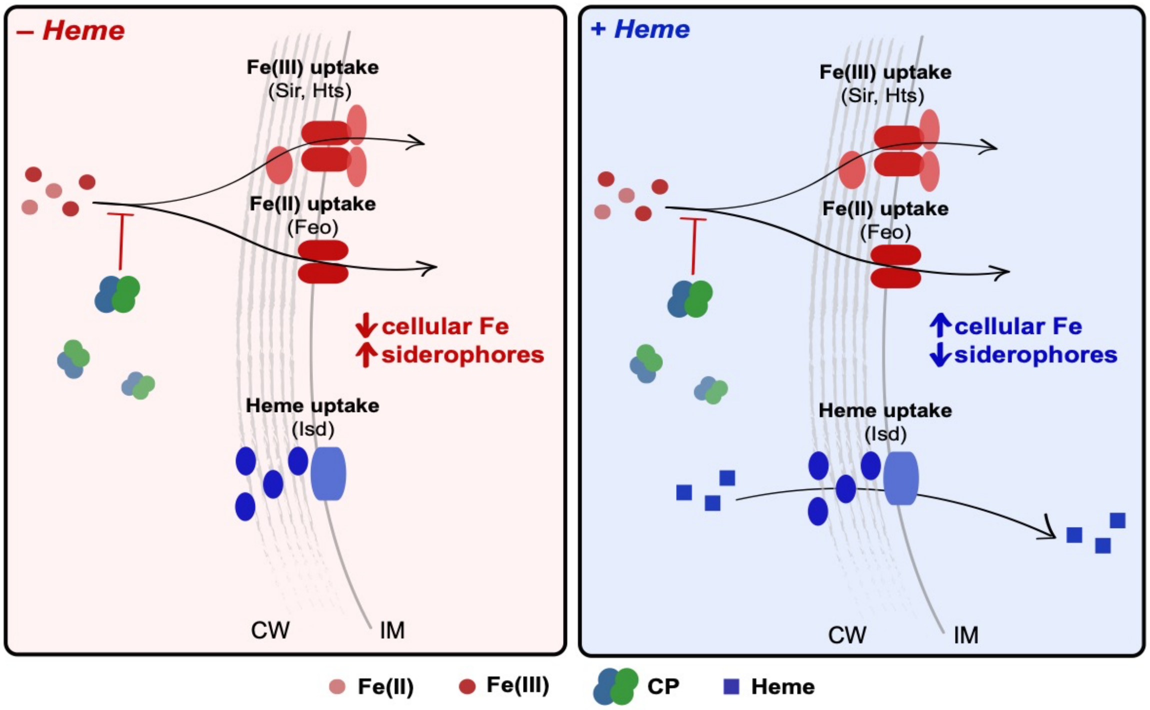

Figure S8. Model for the effect of heme on CP-mediated iron starvation in *S. aureus* based on the current work. (Left) When heme is unavailable, CP inhibits *S. aureus* iron uptake and promotes siderophore production. (Right) When heme is available, *S. aureus* can utilize heme to mitigate the effect of CP on iron uptake, which alleviates its iron starvation responses to CP. CP, calprotectin; CW, cell wall; IM, inner membrane.

Supporting references

1. McDougal, L. K., Steward, C. D., Killgore, G. E., Chaitram, J. M., McAllister, S. K., and Tenover, F. C. (2003) Pulsed-field gel electrophoresis typing of oxacillin-resistant *Staphylococcus aureus* isolates from the United States: establishing a national database. *J. Clin. Microbiol.* **41**, 5113-5120
2. Fey, P. D., Endres, J. L., Yajjala, V. K., Widhelm, T. J., Boissy, R. J., Bose, J. L., and Bayles, K. W. (2013) A genetic resource for rapid and comprehensive phenoype screening of nonessential *Staphylococcus aureus* genes. *MBio* **4**, e00537-12
3. Baba, T., Bae, T., Schneewind, O., Takeuchi, F., and Hiramatsu, K. (2008) Genome sequence of *Staphylococcus aureus* strain Newman and comparative analysis of staphylococcal genomes: polymorphism and evolution of two major pathogenicity islands. *J. Bacteriol.* **190**, 300-10
4. Duthie, E. S., and Lorenz, L. L. (1952) Staphylococcal coagulase; mode of action and antigenicity. *J. Gen. Microbiol.* **6**, 95-107
5. Harik, N. S., Com, G., Tang, X., Melguizo Castro, M., Stemper, M. E., and Carroll, J. L. (2016) Clinical characteristics and epidemiology of methicillin-resistant *Staphylococcus aureus* (MRSA) in children with cystic fibrosis from a center with a high MRSA prevalence. *Am. J. Infect. Control.* **44**, 409-415
6. Harro, J. M., Daugherty, S., Bruno, V. M., Jabra-Rizk, M. A., Rasko, D. A., and Shirtliff, M. E. (2013) Draft genome sequence of the methicillin-resistant *Staphylococcus aureus* Isolate MRSA-M2. *Genome Announc.* **1**, e00037-12
7. Holloway, B. W. (1955) Genetic recombination in *Pseudomonas aeruginosa. J. Gen. Microbiol.* **13**, 572-581
8. Smith, A. D., and Wilks, A. (2015) Differential contributions of the outer membrane receptors PhuR and HasR to heme acquisition in *Pseudomonas aeruginosa*. *J. Biol. Chem.* **290**, 7756-66
9. Rahme, L. G., Stevens, E. J., Wolfort, S. F., Shao, J., Tompkins, R. G., and Ausubel, F. M. (1995) Common virulence factors for bacterial pathogenicity in plants and animals. *Science* **268**, 1899-1902
10. Djapgne, L., Panja, S., Brewer, L. K., Gans, J. H., Kane, M. A., Woodson, S. A., and Oglesby-Sherrouse, A. G. (2018) The *Pseudomonas aeruginosa* PrrF1 and PrrF2 small regulatory RNAs promote 2-alkyl-4-quinolone production through redundant regulation of the *antR* mRNA. *J. Bacteriol.* **200**, e00704-17
11. Reinhart, A. A., Nguyen, A. T., Brewer, L. K., Bevere, J., Jones, J. W., Kane, M. A., Damron, F. H., Barbier, M., and Oglesby-Sherrouse, A. G. (2017) The *Pseudomonas aeruginosa* PrrF small RNAs regulate iron homeostasis during acute murine lung infection. *Infect. Immun.* **85**, e00764-16
12. O'Neill, M. J., and Wilks, A. (2013) The *P. aeruginosa* heme binding protein PhuS is a heme oxygenase titratable regulator of heme uptake. *ACS Chem. Biol.* **8**, 1794-1802
13. Cenciarini, C., Courtois, S., Raoult, D., and La Scola, B. (2008) Influence of long time storage in mineral water on RNA stability of *Pseudomonas aeruginosa* and *Escherichia coli* after heat inactivation. *PLoS One* **3**, e3443
